# Genome-wide codon usage bias analysis in Beauveria bassiana

**DOI:** 10.6026/97320630014580

**Published:** 2018-12-28

**Authors:** Prajna Muthabathula, Saragadam Suneetha, Ratna Grace

**Affiliations:** 1 Department of Botany, Andhra University, Visakhapatnam, Andhra Pradesh, India; 2 2Department of Botany, Dr. V. S. Krishna Government Degree College, Visakhapatnam, Andhra Pradesh, India

**Keywords:** *Beauveria bassiana*, codon usage, translational efficiency, mutational pressure

## Abstract

Codon usage bias analysis allows in identifying the factors that are influencing and contributing to shape the evolution of the organisms. Therefore, it is of interest to analyze 10363 gene sequences from *Beauveria bassiana*. The GC content with 51.50% is higher than the AT content (48.50%) in B. bassiana. The fungal nuclear genes tend to be GC rich and predominantly G/C ending. Codon usage bias exhibited by *B. bassiana* is based on the Relative Synonymous Codon Usage (RSCU) values of 61 sense codons, of which 28 codons are with RSCU value larger than 1. Other factors like Nucleotide composition, mutational pressure and selection also has a role in shaping the codon usage bias. We identified 24 optimal codons that end with G or C. Correlation analysis suggests existence of translational efficiency of amino acids. Based on the GC3s distribution evolution of the *B. bassiana *genes is by the contribution of mutation pressure. ENC may be the major factor in shaping the codon usage bias. This study provides insights into the compositional selection pressure of the genes in *B. bassiana*

## Background

The probability of the codon used for an amino acid over a different codon, which codes for the same amino acid is regarded as codon bias. Different codons that encode the same amino acid are known as synonymous codons. Even though synonymous codons encode the same amino acid it has been shown that for a wide variety of organisms different synonymous codons are used with different frequencies. This phenomenon is termed as codon bias 1[1]. It is found in all eukaryotic and prokaryotic genomes. Codons used more often are referred to as optimized codons or preferred codons. Synonymous codon usage identity may be varying or similar in the genome or among different genes within the genome. Several factors that influence the variations in the codon usage patterns which include genetic drift, mutational pressure and natural selection 2 and these factors are highly responsible for differences in codon usage variations among different organisms. Multiple forms of selection may act resulting in different clusters of synonymous codon usage patterns among genes within the genome 3.

An analysis of genome-wide codon usage bias patterns investigates their consequences and causes and helps in identifying the selective forces that are involved in shaping the evolution of the codon usage patterns, which help in understanding the perspectives of genome biology 4. Codon usage bias of several organisms have been analyzed however, very little is known about codon usage bias in B. bassiana entomo pathogen, belonging to Hypocrealean fungi (cordycepitaceae, Ascomycota) that is used as a potential biopesticide. It is an environmental friendly mycoinsecticide, which is commercially available whose genome was sequenced and light has shed on its differential gene expression and adaptability to different niches 5. It has diversifying roles apart from bio-pesticidal activity, also found as an endophyte both naturally and from inoculated samples and had a role in suppressing plant pathogens 6,7 which makes it more interesting to make further investigations to go through the details of genetic content. The accuracy and efficiency of protein production can be modulated with differences in codon usage while maintaining the same protein sequence 8. Synonymous codon usage patterns identification proves useful in identifying the genes likely under translational selection 9. In this study we analyzed the codon usage bias of B. bassiana. The objectives of the present study are to investigate the presence of codon bias and to identify the preferred codons in the B. bassiana genome and to examine the contribution of influencing factors on the usage of synonymous codons.

## Methodology

**Figure 1:**
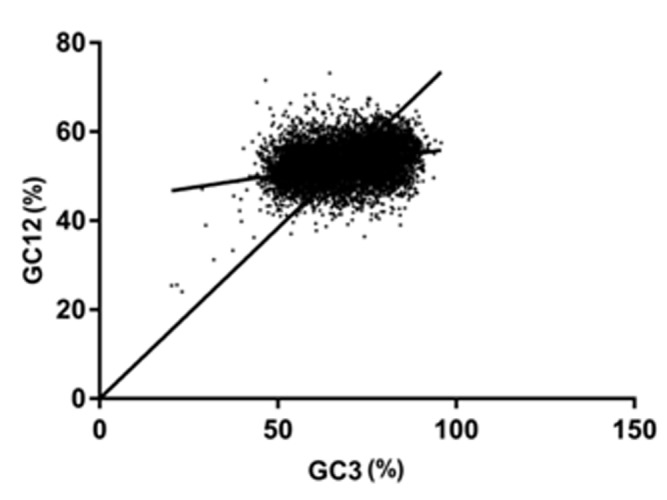
Neutrality plots (GC12 vs. GC3). GC12 is the average value of GC content in the first and second position of the codons. GC3 is the GC content at third position. The solid line is the linear regression of GC12 against GC3, R^2^ = 0.1105, p less than 0.001

The flowchart for methodology is given in Figure 1.

### Sequence data:

The 10363 CDS (Coding domain sequences) dataset of B. bassiana (ASM28067v1) from the whole genome sequence were downloaded from National Centre for Biotechnology Information (NCBI) in FASTA (fasta and fna) format (http://www.ncbi.nlm.nih.gov/genome/).

### Codon usage indices:

Codon usage indices such as GC, GC1, GC2, GC3s, A3s, T3s, C3, and G3s were calculated using CAIcal tool [10]. Relative Synonymous Codon Usage (RSCU) values and Codon Adaptation Index (CAI) values were also calculated 1[10]. General average hydropathicity (GRAVY) and Aromo values (frequency of aromatic amino acids) in the hypothetical translated gene product were also calculated [8].

### Effective Number of Codons (ENC) and ENC plot:

ENC is assessment of non-uniformity of usage within synonymous groups of codons 11. ENC values vary from 20 (extreme bias i.e., only one codon is used for one amino acid) and 61 (random bias i.e., codons used randomly). ENC values were plotted against GC3s values to find out the codon usage bias-influencing factor [11].

### Relative Synonymous Codon Usage (RSCU):

RSCU is defined as the ratio of observed frequency of codons to the expected frequency. If the RSCU value is equal to 1 the codon is not biased and if RSCU value is >1 codon is frequently used.

### Codon Adaptation Index (CAI):

CAI is a measurement of the relative adaptiveness of the codon usage of a gene towards the codon usage of highly expressed genes. CAI values range from 0-1. The higher values indicate a higher-level gene expression as well as codon bias 12.

### Neutrality plot:

The GC content is calculated according to the first, second and third codon positions (GC1, GC2 and GC3 respectively). GC12 is the average of GC1 and GC2 used for the analysis of neutrality plot (GC12 against GC3). Neutrality plot is used to analyze the relationship between GC12 and GC3, and the factors influencing the codon usage bias 13,15.

### Software and statistical analysis:

RSCU, ENC, total G+C genomic content, as well as COA, were calculated by codonW 1.4 version (http://codonw.sourceforge.net/). Values of CAI, GC1, GC2 and GC3 were calculated by CAIcal server (http://genomes.urv.cat/CAIcal/). Statistical analysis was done using R software 3.4.1 version (www.r-project.org) and GraphPad Prism 7.03. Graphs were constructed using GraphPad Prism 7.03 (GraphPad Software Inc., La Jolla, CA, USA).

## Results

### Base composition:

The GC content of 10363 genes distributed between 23.66% to 72.70%, GC12 being distributed between 40.00% to 60.00% (Figure 1). There is a great difference in the GC content of GC2 and GC3, 45.56±5.58 and 66.96±10.44 respectively ( Table 1).

### Neutrality plot:

To characterize the correlation among three positions of GC the neutrality plot is drawn. The relationship between GC12 and GC3 was revealed with neutrality plot (Figure 1). The neutrality plot reveals that the genes of B. bassiana exhibit a wide range of GC3 values, ranging from 20.16% to 95.78%. If a gene is located on the diagonal line with a significant correlation between GC12 and GC3, it indicates that the gene is under neutral selection pressure. The points (genes) were located above the regression curve (bold line) with a slope less than 1, indicating that the natural selection pressure is dominating the composition of coding codons in B.bassiana. GC12 and GC3s showed a significant positive correlation (r= 0.3348, p<0.001). The slope of regression line for all genes was 0.1196, which indicates that the effect of mutation pressure is 11.96% and the influence from other factors is around 88.04%. Effective Number of codons (ENC) and GC3s association The ENC of B.bassiana ranges from 24.68 to 61.00 with an average of 48.02. Among 10363 genes 808 genes exhibited high codon bias (ENC<35), indicating that B.bassiana genes, in general exhibit random codon usage without strong codon bias.

**Figure 2:**
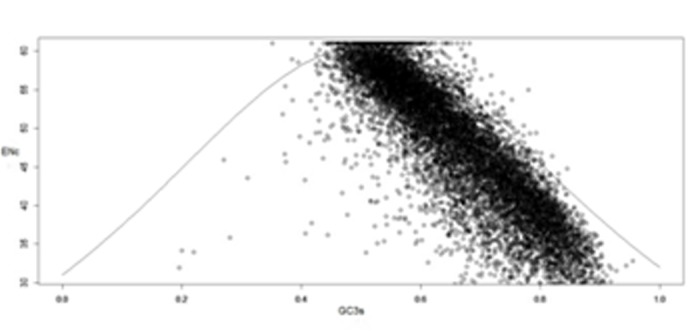
ENC plot between Effective Number of Codons and GC3s. Standard curve represents the respected ENCs to GC3s. The codon usage pattern is affected by other factors besides nucleotide composition as most genes are far away from the standard curve.

An ENC plot was generated to explore the influence of GC3s on codon bias in B.bassiana. If a gene is located on the expected curve, the codons of that gene are no bias. The GC3s distribution was in between 0.4 and 0.99, indicating that B.bassiana mainly evolved by mutation pressure (Figure 2). The distribution of ENC versus GC3s reveals, most of the points with low ENC values lay below the expected curve. This indicates that the mutational pressure and other factors are likely to be involved in determining the selective contribution on codon bias.

### Correlation between codon usage bias, gene length, Hydrophobicity and Aromaticity in B.bassiana:

Correlation between the codon usage indices such as gene length, codon usage bias and hydrophobicity and aromaticity was determined using Spearman correlation analysis ( Table 2). The values showed that the gene length was positively correlated with ENC (r=0.089, p<0.001), suggesting the contribution of gene length to codon usage bias. ENC was negatively correlated with first and second axes (r= -0.934, p<0.001; r= -0.005, p<0.05) and also with GC3 (r= -0.598, p<0.001). GRAVY (General Average Hydropathicity) is negatively correlated with ENC (r= -0.136, p<0.001).

CAI (Codon Adaptaion Index) was negatively correlated with GC1, GC2, G3s and Axis1 (r= -0.364, p<0.001; r= -0.197, p<0.001; r= -0.385, p<0.001 and r= -0.934, p<0.001 respectively) and gene length and ENC were positively correlated with CAI (r= 0.087, p<0.001; r= 0.717, p<0.001) indicating that Nc and gene expression levels contribute to codon usage in a major way. This suggests that ENC may be the major factor in shaping the codon usage in B. bassiana. GRAVY and CAI values showed positive correlation with Aromo (r= 0.362, p<0.001; r= 0.103, p<0.001) indicating that the hydrophobicity, CAI and aromaticity are the most important factors in amino acid usage. This provides a strong evidence for the existence of the selection for translational efficiency of amino acids in B. bassiana.

The number of codons in the high bias dataset was 177993 and number of codons in low bias dataset was 226356. The low and high indicate the top and bottom of the dataset ordered by ENC ratio value respectively. *Optimal codons.

### Optimal codons in Beauveria bassiana

Based on the RSCU values of 61 codons, codon bias exhibited by B.bassiana is weak. Twenty-eight codons were frequently used which showed the high RSCU values such as CGC (RSCU=2.33), GGC (RSCU= 2.16) encoding Arg and Gly respectively. Most frequent codons ended with C or G, such as CUC (RSCU=1.96), GUC (RSCU=1.80), CUG (RSCU=1.66) and GCC (RSCU=1.63) ( Table 3).

Each amino acid has the synonymous codons, the putative optimal codons of B. bassiana are given in ( Table 4). There is a difference in number of synonymous codons for each amino acid. There were 25 optimal codons that end with G or C (G = 10/25, C = 15/25), which suggests the third position in the preferred codons may be related to the GC content. There are two or three optimal codons for each amino acid indicating that the codons were significantly correlated with translation levels.

## Discussion

Codon usage bias is an essential feature of all genomes 14. The GC rich genome of B. bassiana can result in the dominance of the G/C ended codons, where AT rich genome of bacteria show the A/T ended optimized codons 15. GC content close to 50% indicates little overall mutational bias in Aspergillus nidulans, an ascomycete 16, here B.bassiana also has a GC rich genome which too indicates a chance for mutational bias. One of the hypotheses proposed to explain variation of GC content in genome evolution is the �mutational biases hypothesis� is that GC content is driven by heterogenous mutational biases along genomes 17. During the evolution of genomic structures G+C content could be the most important factors 18. Neutrality plot results showed a significant positive correlation in B.bassiana indicating that the effect on the GC contents by the intragenomic GC mutation bias was similar at all three codon positions 19.

To investigate the synonymous codon usage, plotting ENC versus GC3s is an effective strategy 11. ENC may play a role in shaping the codon usage in B.bassiana. Around 7.79% of genes only exhibited codon bias, which indicates that there is random codon usage without strong codon bias in B.bassiana 8. Random codon usage bias in B.bassiana may result due to translational selection as it is responsible for the unequal codon usage of synonymous codons in protein coding genes in a wide variety of organisms 20. The protein produced may not be affected by the synonymous codon chosen but it may relate to the expression of gene 21. In particular, for species of fungi codon usage bias was driven by selection 22-24 and partly genetic interference in the model organism Neurospora crassa 25. Codon usage bias is recognized as a critical factor contributing to gene expression and cellular function with its effects on processes like RNA processing to translation and protein folding 26. Optimal codons were identified by comparing the low and high bias datasets, these codons if significantly correlate with translational levels 19, they would be helpful in designing degenerate primers in order to investigate evolutionary aspects of B. bassiana. B.bassiana exhibit no strong codon usage bias, there is a random codon usage bias. There is a strong evidence for selection of translational efficiency of amino acids and also there is the contribution of mutational pressure and other factors to codon usage bias. The natural selection pressure dominates the codon usage in B.bassiana.

## Conclusion

The present study brings out the codon usage details of entompathogenic fungus Beauveria bassiana. We found no strong codon bias in B.bassiana. Reason for random or selective contribution of codon bias is mutational pressure and other factors like natural selection. There is also influence of translational efficiency of amino acids in shaping codon usage bias. Our analysis forms the footwork of genetic evolutionary aspects of B.bassiana. Further studies may reveal more details relating to the evolution and other molecular aspects of these fungi.

## Figures and Tables

**Table 1:** Base composition, ENc, GRAVY and AROMO of codons of Beauveria bassiana

Class	Genes	Codons	GC%	GC3s	GC1 (%)	GC2 (%)	GC3 (%)	
Total	10363	5182650	57.24±4.97	65.79±10.88	59.19±4.86	45.56±5.58	66.96±10.44	
Class	T3s (%)	C3s (%)	A3s (%)	G3s (%)	Gravy	Aro	ENC	CAI
Total	19.37±5.95	38.55±9.30	13.66±6.12	28.40±6.44	0.32±0.37	0.07±0.02	48.06±8.44	0.62±0.62

**Table 2:** Correlation coefficients between the codon usage indices along the first two major axes and the position of genes

	Length	GC	GC1	GC2	GC3	GC3s	A3s	T3s	C3s	G3s	GRAVY	AROMO	ENC	CAI	AXIS1
GC	-0.153**														
GC1	0.001	0.409**													
GC2	-0.044**	0.299**	0.155**												
GC3	-0.190**	0.595**	0.387**	0.129**											
GC3s	-0.192**	0.598**	0.398**	0.135*	0.999**										
A3s	0.135**	-0.509**	-0.270**	-0.088**	-0.879**	-0.879**									
T3s	0.213**	-0.525**	-0.416**	-0.147**	-0.850**	-0.848**	0.514**								
C3s	-0.140**	0.473**	0.182**	0.124**	0.810**	0.814**	-0.858**	-0.534**							
G3s	-0.074**	0.288**	0.388**	0.026**	0.457**	0.448**	-0.180**	-0.095**	-0.628**						
GRAVY	-0.030**	0.040**	-0.090**	-0.025*	0.096**	0.091**	-0.158**	-0.011	0.154**	-0.067**					
AROMO	0.001	-0.213**	-0.240**	-0.160**	-0.004	-0.016	-0.052**	0.062**	0.047**	-0.078**	0.362**				
ENC	0.089**	-0.774**	-0.254**	-0.090**	-0.596**	-0.598**	0.585**	0.450**	-0.556**	-0.148**	-0.136**	0.01			
CAI	0.087**	-0.920**	-0.364**	-0.197**	-0.608**	-0.608**	0.474**	0.583**	-0.427**	-0.385**	-0.058**	0.103**	0.717**		
AXIS1	-0.078**	0.825**	0.268**	0.091**	0.637**	0.639**	-0.612**	-0.490**	0.593**	0.179**	0.154**	0.016	-0.934**	-0.793**	
AXIS2	0.023**	-0.328**	-0.177**	-0.089**	-0.194**	-0.190**	0.017**	0.332**	0.061**	-0.458**	0.006	0.033**	-0.005	0.530**	-0.043**

**Table 3:** Codon usage of Beauveria bassiana

Amino Acid	Codon	Total count	RSCU	Amino Acid	Codon	Total count	RSCU
Phe	UUU	97,124	1.04	Ser	UCU	63,684	0.92
	UUC	90,092	0.96		UCC	81,713	1.18
Leu	UUA	15,257	0.2		UCA	44,120	0.64
	UUG	61,046	0.8		UCG	84,448	1.22
	CUU	69,405	0.91		AGU	34,859	0.5
	CUC	1,50,168	1.96		AGC	1,06,765	1.54
	CUA	36,366	0.47	Pro	CCU	64,901	0.86
Ile	CUG	1,27,510	1.66		CCC	98,366	1.3
	AUU	1,00,384	1.25		CCA	56,636	0.75
	AUC	1,12,518	1.41		CCG	82,011	1.09
	AUA	27,324	0.34	Thr	ACU	56,368	0.74
Met	AUG	1,15,524	1		ACC	99,999	1.31
Val	GUU	63,597	0.8		ACA	58,385	0.77
	GUC	1,43,919	1.8		ACG	89,814	1.18
	GUA	28,295	0.35	Ala	GCU	1,01,796	0.82
	GUG	83,697	1.05		GCC	2,02,360	1.63
Tyr	UAU	44,250	0.64		GCA	76,891	0.62
	UAC	93,320	1.36		GCG	1,16,536	0.94
Cys	UGU	17,074	0.51	Trp	UGG	74,428	1
	UGC	49,552	1.49	Arg	CGU	44,390	0.82
His	CAU	45,803	0.73		CGC	1,25,865	2.33
	CAC	80,397	1.27		CGA	47,855	0.89
Gln	CAA	77,403	0.73		CGG	40,937	0.76
	CAG	1,33,706	1.27		AGA	36,237	0.67
Asn	AAU	66,104	0.73		AGG	29,101	0.54
	AAC	1,15,356	1.27	Gly	GGU	72,400	0.81
Lys	AAA	68,568	0.57		GGC	1,92,920	2.16
	AAG	1,73,994	1.43		GGA	52,839	0.59
Asp	GAU	1,12,138	0.74		GGG	39,116	0.44
	GAC	1,92,144	1.26	TER	UGA	3,421	0.99
Glu	GAA	1,10,930	0.73		UAA	3,595	1.04
	GAG	1,93,581	1.27		UAG	3,348	0.97

**Table 4:** Optimal codons of Beauveria bassiana

Amino acid	codon	High RSCU	N	Low RSCU	N
Phe	UUU	0.99	3493	1.02	4006
	UUC	1.01	3581	0.98	3865
Leu	UUA	0.02	52	0.52	1782
	UUG	0.21	546	1.14	3947
	CUU	0.37	956	1.25	4325
	CUC*	3.53	9236	1.14	3937
	CUA	0.13	351	0.78	2684
	CUG*	1.74	4564	1.17	4053
Ile	AUU	1.09	3160	1.19	4431
	AUC*	1.85	5373	1.11	4157
	AUA	0.05	159	0.7	2611
Met	AUG	1	3892	1	4860
Val	GUU	0.33	1072	1.11	3542
	GUC*	2.83	9154	1.21	3887
	GUA	0.08	245	0.66	2128
	GUG	0.76	2474	1.02	3260
Tyr	UAU	0.2	531	1.02	2903
	UAC*	1.8	4668	0.98	2809
His	CAU	0.22	434	1.09	3329
	CAC*	1.78	3436	0.91	2780
Gln	CAA	0.31	931	1.01	5088
	CAG*	1.69	4985	0.99	4946
Asn	AAU	0.27	815	1.04	4228
	AAC*	1.73	5282	0.96	3879
Lys	AAA	0.2	885	0.93	4872
	AAG*	1.8	8076	1.07	5585
Asp	GAU	0.29	1419	1.01	6628
	GAC*	1.71	8446	0.99	6509
Glu	GAA	0.33	1498	0.97	6665
	GAG*	1.67	7699	1.03	7095
Ser	UCU	0.47	971	1.16	3849
	UCC*	2.01	4120	0.83	2740
	UCA	0.16	324	1.06	3500
	UCG*	1.52	3103	0.92	3056
	AGU	0.12	243	0.78	2572
	AGC*	1.72	3523	1.26	4164
Pro	CCU	0.41	939	1.04	3239
	CCC*	2.41	5557	0.74	2302
	CCA	0.15	337	1.32	4115
	CCG*	1.04	2409	0.9	2816
Thr	ACU	0.33	862	1.03	3441
	ACC*	2.22	5828	0.86	2873
	ACA	0.21	549	1.23	4104
	ACG*	1.24	3259	0.88	2945
Ala	GCU	0.43	2078	1.07	5240
	GCC*	2.69	13066	0.9	7 4782
	GCA	0.13	616	1.15	5659
	GCG	0.76	3677	0.81	3992
Cys	UGU	0.13	135	0.86	1491
	UGC*	1.87	1916	1.14	1971
Trp	UGG	1	2681	1	3263
Arg	CGU	0.53	858	0.77	1920
	CGC*	4.5	7293	1.11	2778
	CGA	0.18	288	1.35	3373
	CGG	0.47	756	0.76	1916
	AGA	0.13	215	1.21	3024
	AGG	0.19	312	0.81	2022
Gly	GGU	0.47	1675	0.88	3074
	GGC*	3.22	11372	1.3	9 4816
	GGA	0.14	486	1.06	3678
	GGG	0.17	614	0.67	2332
TER	UAA	1.73	299	1.01	175
	UAG	0.76	132	1.01	175
	UGA	0.5	87	0.97	168

**Figure 3:**
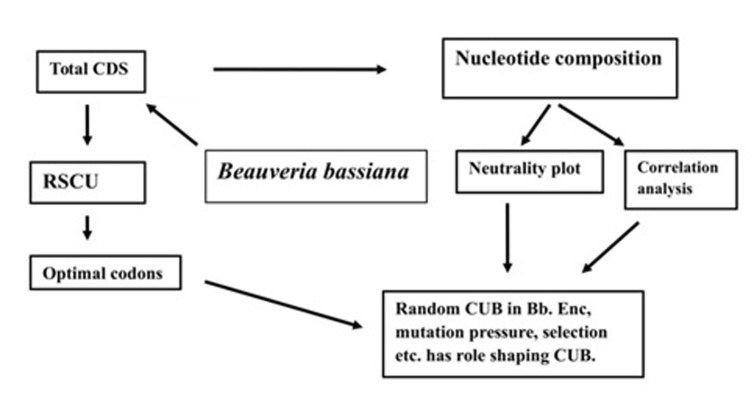
Flowchart for the extraction of CUB is given
